# Effect of Different Extenders on the Oxidative Status and Fertility of Sarda Ram Liquid Semen Stored at 15 °C

**DOI:** 10.3390/antiox14080932

**Published:** 2025-07-30

**Authors:** Valeria Pasciu, Charbel Nassif, Maria Dattena, Sara Succu, Francesca Daniela Sotgiu, Antonello Cannas, Ignazio Cossu, Elena Baralla, Fabrizio Chessa, Fiammetta Berlinguer, Laura Mara

**Affiliations:** 1 Department of Veterinary Medicine, University of Sassari, Via Vienna 2, 07100 Sassari, Italy; succus@uniss.it (S.S.); fdsotgiu@uniss.it (F.D.S.); ebaralla@uniss.it (E.B.); berling@uniss.it (F.B.); 2 Department of Agricultural Sciences, Unit of Animal Science, University of Sassari, Viale Italia 39/A, 07100 Sassari, Italy; c.nassif@studenti.uniss.it (C.N.); cannas@uniss.it (A.C.); 3 Department of Animal Science, Agricultural Research Agency of Sardinia, 07100 Sassari, Italy; mariadattena@gmail.com (M.D.); icossu@agrisricerca.it (I.C.); fchessa@agrisricerca.it (F.C.); lmara@agrisricerca.it (L.M.)

**Keywords:** Ram semen, artificial insemination, livestock, sheep farming, ROS, TEAC, SOD, MDA, mitochondrial membrane potential (MMP), acrosome integrity, ATP

## Abstract

Liquid storage is an important tool used to prolong fresh semen shelf-life while protecting spermatozoa from damage, conserving their overall functionality, and ensuring better fertility than frozen semen from sheep. The increased production of reactive oxygen species (ROS) during sperm storage leads to a decline in sperm quality, particularly with regard to sperm nuclear DNA damage and mitochondrial membrane potential (MMP). This study evaluated the effect of storing Sarda ram semen at 15 °C for 7 h on its redox status, motility, morphology, acrosome integrity, ATP content, mitochondrial potential membrane, and in vivo fertility after artificial insemination. Two different extenders were compared: a lab-made skimmed milk (SM)-based extender and a commercial extender (OviXcell^®^, IMV-Technologies, France). Lower ROS levels in the SM (*p* < 0.001) indicated that its oxidative status was better maintained compared to the commercial extender (CE). Antioxidant defenses (total antioxidant capacity, TEAC; superoxide dismutase, SOD; total thiols) were higher in the SM (*p* < 0.01) than in the CE. SM also had higher MMP (*p* < 0.05), acrosome integrity (*p* < 0.05), ATP content (*p* < 0.01), and in vivo fertilizing capacity (*p* < 0.05) compared to the CE, which indicated higher semen quality. In conclusion, the SM extender, while maintaining a better oxidative/antioxidant balance, ensured higher semen quality after 7 h of storage at 15 °C in vitro compared to the CE.

## 1. Introduction

Artificial insemination (AI) is a technique widely used in livestock to quickly release the reproductive potential of genetically superior males [1,2]. Extending spermatozoa lifespan is crucial for the application of AI as it provides breeders with a necessary time-window to plan and execute inseminations effectively. By decreasing the storage temperature, the energy consumption of sperm is significantly reduced, thus conserving their functional capacity and prolonging the duration for which they remain capable of fertilization.

However, the dilution of seminal plasma, an important source of antioxidants, predisposes stored sperm to oxidative stress, as has been shown for bull semen [3].

The excessive production of reactive oxygen species (ROS) might have serious implications on sperm structure and functionality because spermatozoa are particularly susceptible to damage induced by ROS [4]. An increase in ROS production has been linked to a proportional alteration in mitochondrial membrane potential (MMP) in spermatozoa from different species [5,6]. Furthermore, excess ROS inhibit sperm capacitation [1,7], increase sperm nuclear DNA damage [6] and peroxidation products such as malondialdehyde (MDA) [6], and decrease MMP [8] and sperm motility [9]. The consequent altered mitochondrial function decreases sperm motility and reproductive capacity through oxidative damage [5,9]. For this reason, the protection of spermatozoa from ROS during storage is pivotal for ensuring the maintenance of their functionality and fertilizing ability. Extenders used to dilute semen before storage can be crucial in this regard [4,6,10]. One of the most diffused dilutors for ram semen are skimmed-milk-based extenders (SM). It is worth emphasising, however, that the viability and fertility of ram spermatozoa after storage for 8–16 h were better at 15 °C than at 5 °C [11,12,13]. However, this semen needs to be used within 8–10 h after dilution to guarantee good fertility [14]. Some researchers have also used a commercial extender (CE) to improve semen preservation for rams (OviXcell^®^ (IMV Technology, L’Aigle, France) [15].

Given these premises, the primary objective of the current study was to evaluate the spermatozoa oxidative status after 7 h of liquid storage at 15 °C using two extenders (SM and CE). We measured a panel of other functional parameters that included acrosome integrity, ATP content, mitochondrial membrane potential, and in vivo fertilizing ability after AI. The oxidative status in the sperm cells was assessed by quantifying ROS level, the activity of superoxide dismutase (SOD), Trolox equivalent antioxidant capacity (TEAC), total thiols, and malondialdehyde (MDA). Both the ROS and functional parameters were measured in fresh raw semen upon collection and after liquid storage. In addition, the total and progressive motility and normal morphology were assessed after dilution and cooling at 15 °C (0 h) and then again after 7 h of storage.

## 2. Materials and Methods

### 2.1. Experimental Design

The analyses listed and described below were performed at two different time points: on raw semen upon collection, and on liquid stored semen—diluted with two different extenders–after 7 h at 15 °C. Each analysis was replicated 3 times. Motility and morphology were also evaluated immediately after dilution and cooling at 15 °C and after 7 h of storage at the same temperature. The duration of the storage time (7 h) was based on the time until the semen arrived at the intended farm and the insemination of the hormonally synchronized ewes.

### 2.2. Collection, Assessment of Raw Semen, and Dilution

Three Sarda-breed adult rams (4–6 years old), of proven high semen quality, were used in the experiment. Semen for all the analyses was collected using an artificial vagina twice: at the end of May and end of July at the Genetic Centre (ASSO.NA.PA) in the research institute Agris Sardegna–Bonassai. These dates were chosen to provide representative samples from the start and the end of the AI period in Sardinia.

The ejaculates were evaluated using Computer-Assisted Sperm Analysis (CASA) with the Ceros II system by Hamilton-Thorne (Bioscience, Beverly, MA, USA). After raw semen evaluation, the ejaculate from each ram was divided into two aliquots and diluted to a concentration of 1.6 × 10^9^ spermatozoa/mL, employing two different extenders at 38 °C: SM [16,17] and CE.

### 2.3. Motility and Morphology

Once the temperature of 15 °C was reached (0 h), the sample was evaluated using CASA and then packaged in 0.25 mL straws, sealed, and maintained at 15 °C. The semen was re-evaluated for all parameters after 7 h of storage. Five fields were selected for analysis. The total motile sperm (TM, all the spermatozoa that moved), progressive motility (PM, spermatozoa having a linear trajectory), and normal morphology (NM, those that had no morphological abnormalities) were evaluated.

### 2.4. Acrosome Integrity

Acrosome integrity was evaluated using fluorescein an isothiocyanate-labelled Pisum sativum agglutinin (FITC-PSA) (5 μg/mL) and propidium iodide (PI; 14 μg/mL) as previously described [18]. The results were reported as the percentage of viable spermatozoa with intact acrosomes, counting 200 spermatozoa per slide.

### 2.5. Mitochondrial Membrane Potential (MMP)

The mitochondrial membrane potential (MMP) was measured by staining spermatozoa with the probe 5,5,6,6-tetrachloro-1,1,3,3-tetraethylbenzimidazolyl carbocyanine iodide (JC-1). The JC-1 was dissolved in dimethyl sulfoxide (5 mg/mL and 7.7 mM) and added to the cells (30 × 10^6^ spermatozoa/mL) at a final concentration of 1 µM for 15 min at 37 °C. This dye differentially labelled the mitochondria according to their membrane potential [19,20]. The fluorescence of the sperm with JC-1 was measured in two ways: a fluorescent measurement and microscopic staining, as explained below.

#### 2.5.1. Fluorescent Measurement of the Sperm with JC-1

The fluorescent emission of the sperm with JC-1 was measured as previously described [20,21,22] using a FLUOstar Omega microplate reader (BMG LABTECH, Ortenberg, Germany). All results are expressed as the ratio of relative fluorescence units (RFU) of rhodamine/fluorescein per number of spermatozoa.

#### 2.5.2. Microscopic Staining of the Sperm with JC-1

Microscopic staining of the sperm with JC-1 was evaluated as described by Agnihotri S.K. et al. [21] using a Diaphot (Nikon, Tokyo, Japan) epifluorescence microscope.

### 2.6. ROS Assay

ROS production in the ram spermatozoa was measured using 2′,7′-dichlorodihydrofluorescein diacetate (H_2_DCF-DA) at 2 μM as previously described [6]. All fluorescence measurements (RFU mean/n° spermatozoa ± SD) (excitation/emission at 485/535 nm) were corrected for background fluorescence and replicated four times.

### 2.7. Sample Preparation for the Assay SOD, TEAC, Total Thiols, and MDA

We centrifuged 200 µL of semen samples (250 × 10^6^ spermatozoa/mL) at 1500× *g* for 10 min. The resulting pellets were treated for cellular extraction with PBS containing 0.1% Triton X-100 (500 μL of PBS-Triton X-100 0.1% for every 250 × 10^6^ total spermatozoa). Superoxide dismutase (SOD), Trolox-equivalent-antioxidant-capacity (TEAC), total thiols, and malondialdehyde (MDA) concentration were measured in cellular extracts [6,18].

SOD activity was measured enzymatically as described previously [6,18] at 470 nm [23].

TEAC was determined using the method described previously [18,24]. The total thiols were assayed using Ellman’s Reagent [25] and 5,5-dithiol-bis-(2-nitrobenzoic acid) dissolved in PBS, as described previously [26]. MDA, one of the low-molecular-weight end-products of lipid peroxidation, was determined using the thio-barbituric-acid-TBARS method, as described previously [18] and according to the TBA test described [27] at 535 nm.

### 2.8. ATP Assay

Intracellular ATP concentration was determined by an enzymatic assay [4] at 340 nm at 37 °C.

### 2.9. Artificial Insemination

A total of 133 ewes were synchronized using flugestone acetate-impregnated sponges (Chronogest^®^ CR 20 mg, MSD animal health) for 14 days + 400 IU PMSG (Folligon^®^, MSD animal health) at sponge removal and inseminated after 55 h by depositing the semen at the first cervical fold. The inseminations occurred on different dates from the end of May till the end of July at the research center at Agris Sardegna in two locations, Bonassai and Monastir, by the same inseminator. Sixty-three ewes were inseminated with SM-diluted semen, and seventy ewes were inseminated with CM-diluted semen. A semen dilution medium for each ram was randomly allocated on the day of collection, alternating media between days.

### 2.10. Statistical Analysis

The effects of storage time and diluent type (SM vs. CE) on the sperm quality parameters, including normal morphology and total and progressive motility, were evaluated using two-way ANOVA. For the fertility and prolificacy results, a chi-square test was performed. For the other parameters studied, after checking the normality and homogeneity of the variance assumptions, differences between the experimental groups were analyzed by one-way ANOVA (SigmaPlot 15.0). The data are expressed as means ± SEMs of at least three replicates. Statistical significance was accepted at *p* < 0.05.

## 3. Results

### 3.1. Motility and Morphology

There were no significant differences between all parameters (TM, PM, and NM) for the raw semen collected at the end of May and July (*p* > 0.05) (Table 1).

When comparing the diluents and storage time, the results indicated that NM was not significantly affected by time (0 vs. 7 h; *p* = 0.376) nor by diluent (*p* = 0.063; Table 2). No significant interaction between time and diluent was observed (*p* = 0.300).

In contrast, TM was significantly reduced after 7 h of storage (*p* = 0.0002), while no significant differences were observed between the two diluents (*p* = 0.569), nor was there a significant interaction (*p* = 0.598). PM followed a similar trend, with a significant decrease over time (*p* = 0.0156), no significant effect of the diluent (*p* = 0.449), and no time–diluent interaction (*p* = 0.885).

### 3.2. Acrosome Integrity

**Figure 1:**
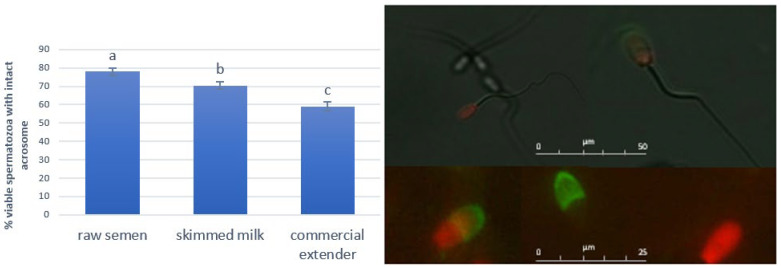
Effect of SM and CE on acrosome integrity for ram spermatozoa after 7 h storage at 15 °C. The lowercase letters indicate significant statistical differences between the groups (*p* < 0.05 a vs. b and b vs. c; *p* < 0.001 a vs. c). The microscopic image shows spermatozoa with intact acrosomes (no color, i.e., no fluorescent staining), a live spermatozoon with a damaged acrosome shown in green (FITC-PSA positive), a dead spermatozoon with a damaged acrosome shown in green and red (PI and FITC-PSA positive), and a dead spermatozoon shown in red (PI positive and FITC-PSA negative).

As shown in Figure 1, the percentage of viable spermatozoa with intact acrosomes decreased after storage as compared to the raw semen (*p* < 0.05) but maintained higher values in SM spermatozoa compared to the CE ones (*p* < 0.001).

### 3.3. Mitochondrial Membrane Potential (MMP)

**Figure 2:**
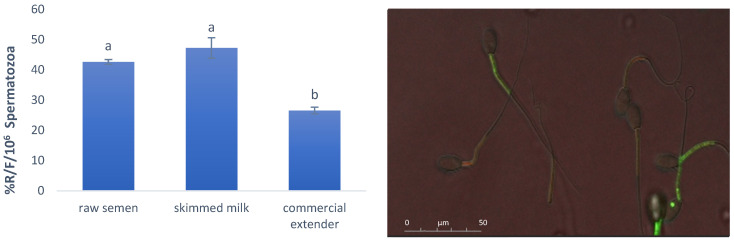
Effect of SM and CE on mitochondrial membrane potential (MMP) for ram spermatozoa after 7 h at 15 °C. The lowercase letters indicate significant statistical differences between the groups (*p* < 0.05 a vs. b, b vs. c, and a vs. c). The microscopic image shows spermatozoa with green fluorescence (fluorescein) that had low MMP and orange fluorescence (rhodamine) for those with vital and high MMP. Mitochondrial depolarisation was indicated by a decrease in the orange/green fluorescence intensity ratio.

No difference was found for the MMP between the raw semen and the semen stored with the SM extender for 7 h at 15 °C (Figure 2). In contrast, the sperm diluted with the CE showed a significant decrease in MMP after storage (*p* < 0.05).

### 3.4. ROS Assay

**Figure 3:**
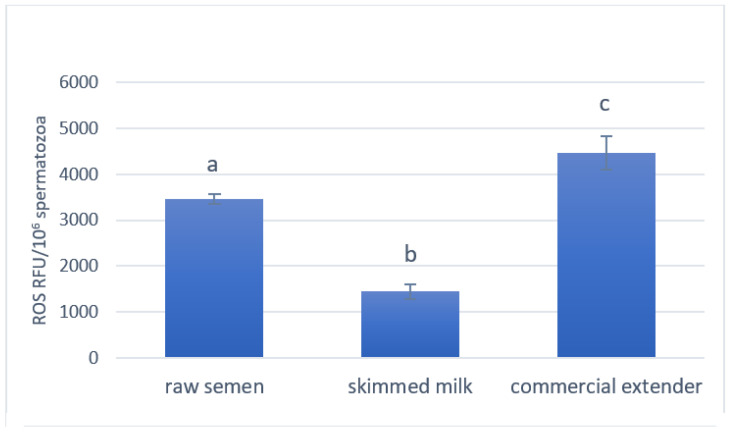
Ram spermatozoa ROS production in the raw semen and after 7 h at 15 °C with SM and CE. The lowercase letters indicate significant statistical differences between the groups (*p* < 0.001 a vs. b and b vs. c; *p* < 0.05 a vs. c).

After 7 h, ROS production significantly decreased in the presence of SM (*p* < 0.001) and increased with CE (*p* < 0.05) compared to the raw semen (Figure 3).

### 3.5. SOD, TEAC, Total Thiols, and MDA Assay

**Figure 4:**
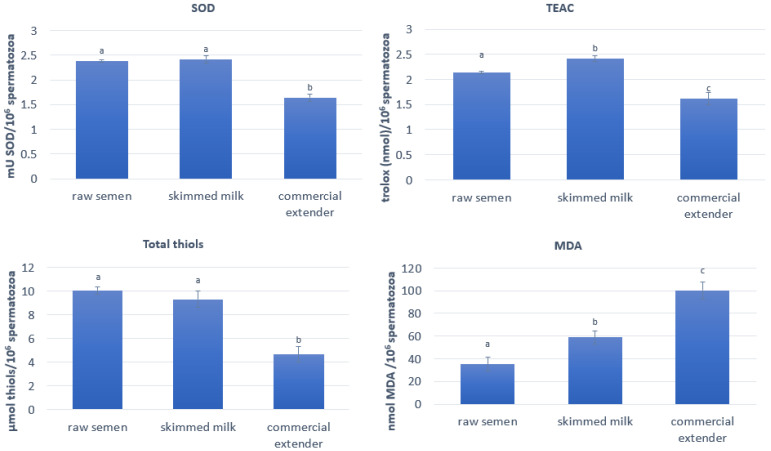
Ram spermatozoa SOD, TEAC, total thiols, and MDA assay for the raw semen and after 7 h storage at 15 °C with the SM and CE. The lowercase letters indicate significant differences between the groups (SOD *p* < 0.01 a vs. b; TEAC *p* < 0.05 a vs. b and *p* < 0.01 a vs. c and b vs. c; total thiols *p* < 0.01 a vs. b; MDA *p* < 0.01; a vs. b, a vs. c, and b vs. c).

In the SM-diluted semen, after 7 h of storage at 15 °C, SOD activity (Figure 4) and the total thiols intracellular concentrations (Figure 4) were maintained at levels comparable to those found in the raw semen. On the other hand, both variables significantly decreased (*p* < 0.01) in the semen diluted with the commercial extender. TEAC (Figure 4) increased significantly (*p* < 0.05) during storage with the SM extender, while it significantly decreased (*p* < 0.01) with the commercial extender, as compared to the raw semen. Regarding MDA (Figure 4), it significantly increased (*p* < 0.01) with both extenders, but the increase was higher for the CE than for the SM.

### 3.6. ATP

**Figure 5:**
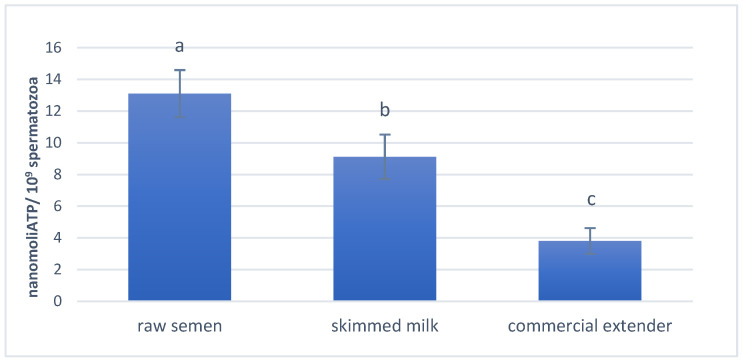
Ram spermatozoa ATP assay for raw semen and after 7 h storage at 15 °C for the skimmed milk (SM) and commercial extender OviXcell^®^ (CE). The data were obtained using an enzymatic assay and are expressed as nanomol ATP/10^9^ spermatozoa. The lowercase letters indicate significant differences between the groups (*p* < 0.01).

After 7 h of storage, ATP intracellular concentrations (Figure 5) decreased significantly for both extenders compared to the raw semen (*p* < 0.01), with a greater decrease in the CE than in the SM (*p* < 0.001).

### 3.7. Fertility After Artificial Insemination

The comparison of fertility between the diluents revealed a statistically significant difference (χ^2^ = 4.46, *p* = 0.035), with ewes inseminated with the semen diluted in SM showing higher fertility rates (42.9%) compared to those inseminated with the CE (23.9%; Table 3). Similarly, a significant association was found between fertility and ram used (χ^2^ = 5.89, *p* = 0.053), with variability observed across Ram1 (23.1%), Ram2 (42.4%), and Ram3 (43.8%). A significant association was also observed between fertility and farm (χ^2^ = 5.79, *p* = 0.016), with ewes in Monastir showing notably higher fertility rates (42.1%) compared to those in Bonassai (20.4%; Table 4). However, when fertility was analyzed within diluents, no significant differences were found between the farms for either the SM (χ^2^ = 2.15, *p* = 0.143) or CE (χ^2^ = 1.14, *p* = 0.286), despite trends suggesting higher fertility for ewes in in Monastir within both groups.

Regarding prolificacy, there was no significant effect of diluent type on the number of lambs born (F = 0.02, *p* = 0.891). The mean prolificacy was comparable between the groups (SM: 1.33, CE: 1.31, Table 3).

## 4. Discussion

The present study showed that the extender used for semen dilution during liquid storage at 15 °C has a significant impact on semen oxidative status, which, in turn, affects semen function and fertilizing ability after AI.

Sperm total, progressive motility, and normal morphology did not significantly change within the two extenders. This suggests that both SM and CE provide similar environments for sperm total motility during 7 h of conservation [28,29]. However, progressive motility showed a significant decrease over time for both extenders, which can be explained by energy depletion or membrane integrity damage [2].

In our study, decreases in intracellular ATP concentrations were observed in both groups, as has been reported by other authors [2].

ATP is one of the main sources of energy to be used for motility [30,31], and the higher ATP concentration found in our study for the SM was associated with lower ROS production and higher MMP when compared with the CE. This was in accordance with the literature [21,31,32,33] and also for boar semen [34]. Indeed, Hennin et al. [35] reported that sperm quality and energy status, measured as ATP intracellular levels, remained stable in samples stored at 25 °C and 17 °C, while they decreased after cooling and storage at 15–10 °C or 5 °C as a consequence of the provoked cold shock in a subset of sperm, with subsequent losses in viability and motility.

Li Q. et al. [36] confirmed that in boar semen refrigerated at 17 °C, acrosome integrity always decreased when compared to the control, and MDA increased from the first day of storage.

It is well known that ROS cause extensive damage, including reduced mitochondrial membrane integrity in ram semen [37]. Mitochondrial dysfunction and high ROS production are associated with a decrease in ATP production, thereby contributing to semen infertility [21,32]. High MMP values in spermatozoa are considered necessary for maintaining chromatin integrity, normal morphology, good motility, and the induction of acrosome reaction; to the contrary, a membrane potential drop is an index of mitochondrial dysfunction [31,33]. MMP is negatively correlated with high ROS generation [32].

Li et al. [34], for boar semen, reported that an alleviation in the parameters of oxidative stress, MMP decline, and ATP depletion improved acrosomal membrane integrity and semen storage efficiency at 17 °C. These findings were confirmed by our study for ram semen.

Indeed, the better oxidative/antioxidative balance found for the SM was related to the higher percentage of viable spermatozoa with intact acrosomes found in this group when compared to the CE.

ROS are also involved in spermatozoa capacitation and acrosomal reaction and with the fusion of spermatozoa and oocytes, and so when they are at high levels, they have been associated with sperm infertility in different animal species [38,39]. In accordance with their better oxidative status, the spermatozoa stored in the SM outperformed those stored in the CE in terms of fertility rates after AI.

These results were consistent with previous studies highlighting the protective effects of milk-based extenders on spermatozoa during storage at 15 °C [40] and at 4–5 °C [29,41,42,43,44]. However, the prolificacy results indicated no significant impact of diluent type on the number of lambs born per ewe. So, even if the diluent influenced conception success, it did not affect prolificacy, which might be more closely tied to genetic and physiological factors of a ewe rather than to semen extenders [12].

In conclusion, our findings suggest that SM provides a more supportive environment for preserving sperm viability and function compared to CE, as confirmed by the higher fertility rates obtained after AI.

Further and more detailed studies are planned to investigate the component of the dilution medium that provides better oxidative protection in semen, as well as interaction studies that include other variables affecting fertility rates.

## Figures and Tables

**Table 1:** Descriptive undiluted raw semen CASA analysis for sperm normal morphology and total and progressive motility percentages ± SE for all three rams for the semen collection from May and July.

Raw Semen	May	July
Normal Morphology (%)	92.33 ± 1.20	91.33 ± 2.73
Total Motility (%)	93.00 ± 2.65	91.33 ± 1.67
Progressive Motility (%)	73.33 ± 6.06	73.00 ± 2.00

**Table 2:** Effect of diluent type (SM and CE) and incubation time on sperm NM, TM, and PM for ram semen ± SE after dilution and reaching 15 °C (0 h) and after 7 h of incubation. *** *p* < 0.0001; * *p* < 0.01.

Diluted Semen	0 h SM	0 h CE	7 h SM	7 h CE	p_Time	p_Diluent	p_Interaction
Normal Morphology (%)	96 ± 0.62	96.7 ± 0.49	95.9 ± 1.26	98.2 ± 0.34	0.376	0.0627	0.2996
Total Motility (%)	92.3 ± 1.88	90.2 ± 2.32	82.6 ± 2.24	82.6 ± 2.69	0.0002 ***	0.5687	0.5981
Progressive Motility (%)	77.1 ± 3.78	75 ± 2.9	68.3 ± 3.78	65.2 ± 3.94	0.0156 *	0.449	0.8854

**Table 3:** Percentage fertility ± SE by medium, individual ram, and prolificacy by medium. * *p* < 0.01.

	SM	CE	Ram 1	Ram 2	Ram 3	Prolificacy	
						Milk	OviXcell^®^
Percentage (%)	42.9 ± 6.2	23.9 ± 5.2	23.1 ± 5.2	42.4 ± 8.6	43.8 ± 8.8	1.33 ± 0.06	1.31 ± 0.06
Chi2	4.46		5.89				
df	1		2				
*p*_value	0.035 *		0.053			0.891	

**Table 4:** Percentage fertility ± SE by farm location in general and within each diluent. * *p* < 0.01.

	Bonassai	Monastir	Bonassai	Monastir	Bonassai	Monastir
			Milk		OviXcell^®^	
Percentage (%)	20.4 ± 5.5	42.1 ± 5.7	26.3 ± 10.1	50 ± 7.5	17.1 ± 6.4	31.2 ± 8.2
Chi2	5.79		2.15		1.14	
df	1		1		1	
*p*_value	0.016 *		0.143		0.286	

## Data Availability

Detailed data are available upon request.

## Abbreviations

The following abbreviations are used in this manuscript:

AI	Artificial insemination
MMP	Mitochondrial membrane potential
ROS	Reactive oxygen species
OS	Oxidative stress
MDA	Malondialdehyde
SOD	Superoxide dismutase
TEAC	Trolox equivalent antioxidant capacity
SM	Skimmed milk
CE	Commercial extender (OviXcell^®^)
CASA	Computer-assisted sperm analysis
RFU	Relative fluorescence units
NM	Normal morphology
H_2_DCF-DA	2′,7′-dichlorodihydrofluorescein diacetate
TM	Total motile sperm
PM	Progressive motility
